# Forgotten Biliary Plastic Stents: Complications, Management, and Clinical Outcomes

**DOI:** 10.3390/medicina60081258

**Published:** 2024-08-02

**Authors:** Mohamed A. Elsebaey, Mohamed Elsayed Enaba, Heba Elashry, Tamer A. Elbedewy, Ahmed Mohamed El Nakib, Ahmed A. Elhadidy, Mohamed Elsayed Sarhan, Waleed Elrefaey, Rasha Youssef Hagag, Abdullah Mohammed Alqifari, Assem Mohamed Elsokkary, Mohamed Abd Allah Alabd, Abdulrashid Onimisi Abdulrahim, Yousry Esam-Eldin Abo-Amer, Ashraf Rafat Abo-Elfetoh, Mohammad Shaaban Mahfouz, Mohamed Saleh, Ahmed Abdelhaleem Mohamed, Amro Abdelaziz Mohammed Ismail

**Affiliations:** 1 Internal Medicine Department, Faculty of Medicine, Tanta University, Tanta 31527, Egypt; mohamedelsebaey79@gmail.com (M.A.E.); m.enaba@hotmail.com (M.E.E.); tamerelbedewy@yahoo.com (T.A.E.); ahmed.elhadedy@med.tanta.edu.eg (A.A.E.); aorta2025@yahoo.com (M.E.S.); dr.waleedelrefaey@gmail.com (W.E.); rasha.hagag@med.tanta.edu.eg (R.Y.H.); 2 Tropical Medicine Department, Faculty of Medicine, Tanta University, Tanta 31527, Egypt; hebae92@gmail.com; 3 Tropical Medicine Department, Faculty of Medicine, Mansoura University, Mansoura 35516, Egypt; 4 Gastroenterology Department, King Fahad Specialist Hospital, Buraydah 52366, Saudi Arabia; dr.abdullahmq@gmail.com; 5 Internal Medicine Department, Mansoura New General Hospital, Mansoura 34008, Egypt; assemmelsokkary@gmail.com; 6 Gastroenterology, Hepatology and Infectious Diseases Department, Red Crescent Hospital, Tanta 66232, Egypt; mohamedelabd009@gmail.com; 7 Gastroenterology/Internal Medicine Department, Federal Medical Centre Keffi, Keffi 961101, Nasarawa State, Nigeria; abdulrashid.aoa@gmail.com; 8 Hepatology, Gastroenterology and Infectious Diseases Department, Mahala Hepatology Teaching Hospital, El-Mahalla el-Kubra 31951, Egypt; dryousryaboaamer@hotmail.com (Y.E.-E.A.-A.); ashrafrafat@gmail.com (A.R.A.-E.); 9 Hepatology, Gastroenterology and Infectious Diseases Department, Ahmed Maher Teaching Hospital, Cairo 11638, Egypt; mashhap8@gmail.com; 10 Internal Medicine Department, National Hepatology and Tropical Medicine Research Institute, Cairo 11638, Egypt; mohamedsaleh20@gmail.com; 11 Tropical Medicine Department, National Hepatology and Tropical Medicine Research Institute, Cairo 11638, Egypt; drhalim2011@gmail.com; 12 Internal Medicine Department, Faculty of Medicine, Cairo University, Giza 12613, Egypt; amroabdelaziz@kasralainy.edu.eg

**Keywords:** forgotten biliary stents, acute cholangitis, stent clogging, stent migration

## Abstract

*Background and Objectives*: Endoscopic biliary plastic stenting is a safe and effective temporary therapeutic modality used in various benign biliary disorders. Long-term indwelling stents for more than one year without retrieval are termed “forgotten biliary stents”. In clinical practice, the forgotten stents are underestimated and the majority of data were obtained from case reports. The aim of this study was to determine the forgotten-biliary-plastic-stent-related complications, their management, and the patients’ clinical outcomes. *Materials and Methods*: This retrospective study was performed at three hospitals during the period from January 2021 to December 2023. In total, 577 patients with biliary plastic stents—inserted for a variety of benign biliary conditions—were included. They were divided into two groups, as follows: group 1 included 527 patients who had biliary stents removed within 3 months, and group 2 included 50 patients with biliary stents retrieved after one year of their deployment. The stent-related complications (e.g., acute cholangitis, stent clogging, distal stent migration, new common bile duct (CBD) stone formation, and proximal stent migration) and the endoscopic management success rate were evaluated. *Results*: Irretrievable CBD stones were the main indication for biliary plastic stenting in both groups. The stent-related complications, number of endoscopic sessions, and hospital admissions were significantly higher in the patients with forgotten biliary stents than those with stent removal within 3 months. All the study patients were successfully managed endoscopically with uneventful outcomes. *Conclusions*: Based on this retrospective study, non-adherence to the endoscopists’ instructions is the main reason for retained biliary stents for more than one year. The patients with forgotten stents had significantly higher complication rates, a higher number of endoscopic sessions, and a higher number of hospital admissions than those with stents that were retrieved in the scheduled time. All patients were managed endoscopically with a technical success rate of 100%, and with no complication-related mortality.

## 1. Introduction

Endoscopic retrograde cholangiopancreatography (ERCP) is a complex procedure utilized to investigate and manage various pancreatic–biliary disorders, most commonly for extracting common bile duct (CBD) stones. ERCP entails multiple risks and can lead to major complications [1]. Endoscopic biliary plastic stenting is a well-established and widely accepted modality for the management of different benign biliary disorders such as irretrievable CBD stones, benign biliary strictures, and post-cholecystectomy biliary leaks [2,3]. Biliary plastic stents are indicated when short-term relief of biliary obstruction is intended [4]. Despite their widespread use in clinical practice, being inexpensive and easy to be removed or replaced, biliary plastic stents are prone to clogging a few days or several months later [5,6,7]. Therefore, society guidelines recommend that biliary plastic stents are removed or exchanged within 3 months from the index procedure [8,9]. The patient’s adherence to the stent follow-up scheduled time is vital. Biliary plastic stents retained for more than one year from the index ERCP without retrieval are defined as “forgotten biliary stents”. These forgotten stents can result in major consequences, including acute cholangitis, stent occlusion, proximal and distal migration, and biliary stone and giant stentolith formation [10,11,12]. Acute cholangitis occurs due to biliary blockage that results in bile stasis, infection, and the translocation of the microorganisms or endotoxins from the infected bile into systemic circulation. The diagnostic criteria and severity grading of acute cholangitis were based on the Tokyo guidelines 2018 [13]. To the best of our knowledge, there are few published studies about forgotten biliary plastic stents and most data were obtained from case reports. In clinical practice, the forgotten stents are often overlooked, underestimated, and seldom reported. This retrospective study was conducted to determine forgotten-biliary-plastic-stent-related complications, their management, and the patients’ clinical outcomes.

## 2. Patients and Methods

### 2.1. Study Design

This retrospective study was carried out during the period from January 2021 to December 2023 at the Internal Medicine Department of Tanta University Hospital, Ibn Sina Specialized Hospital and American Hospital of Tanta, Egypt. During the study period, 1968 ERCP procedures were performed. Of them, 527 patients had biliary plastic stents removed within 3 months (group 1), and 50 patients had biliary plastic stents retrieved after one year of their deployment (group 2); both groups were included in the present study. The current study was approved by the local research ethical committee of the faculty of medicine, Tanta University, Egypt (approval code: 36264PR292/8/23; approval date: (9 August 2023), and conformed to the ethical principles for medical research of the Helsinki Declaration 1975 and its subsequent amendments (1983).

### 2.2. Inclusion Criteria

The inclusion criteria were patients aged more than 18 years, with endoscopic biliary plastic stents retrieved within 3 months, or after one year of their deployment. These stents were previously deployed in these patients for different indications including irretrievable CBD stones, post-cholecystectomy biliary leak, Mirizzi syndrome, and benign CBD strictures.

### 2.3. Exclusion Criteria

Patients with ERCP procedures without biliary plastic stenting, and those with biliary plastic stents retained for a period of more than 3 months and up to 12 months were excluded from this study. Patients with indwelling biliary metal stents and those with malignant biliary obstruction were also ruled out.

### 2.4. Data Collection

The following data were obtained from the patients’ medical files and were analyzed retrospectively: age, gender, comorbidities, index ERCP findings and plastic stenting indications, type and duration of the deployed stents, presenting clinical features, results of laboratory investigations, and the details of the ERCP procedures for management of the biliary plastic stents and their related complications. The reasons for forgotten biliary stents were obtained by conducting telephone interviews with the patients.

### 2.5. Endoscopic Management of the Biliary Plastic Stents and Their Related Complications

**Figure 1:**
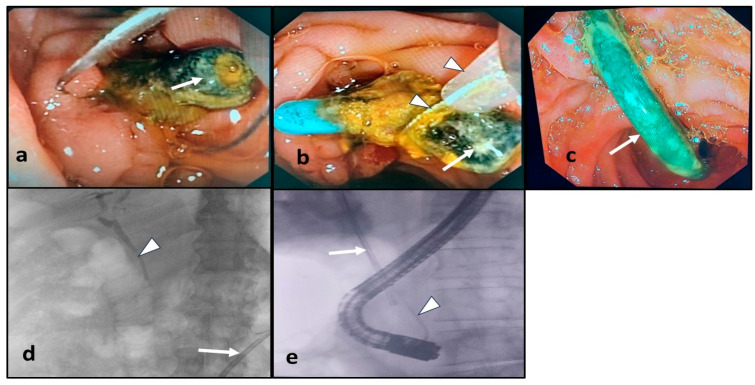
Endoscopic management of the forgotten-biliary-plastic-stent-related complications. (**a**) Endoscopic image of a clogged biliary plastic stent in situ (arrow); (**b**) endoscopic image during retrieval of the clogged stent (arrow) using a snare (arrow heads); (**c**) endoscopic image of a distally migrated biliary plastic stent (arrow), which was accessible for endoscopic retrieval; (**d**) fluoroscopic visualization of a distally migrated biliary plastic stent (arrow), which was inaccessible for endoscopic retrieval, and presence of cholangiogram (arrow head); (**e**) fluoroscopic visualization of a proximally migrated biliary plastic stent (arrow) and the guidewire, which was introduced alongside the stent (arrow head).

The endoscopic procedures were performed under deep sedation using intravenous midazolam and propofol. Some patients received intravenous hyoscine N-butylbromide to minimize intestinal perstalsis. All ERCP procedures were carried out using video duodenoscopes (Olympus TJF-Q180V, Tokyo, Japan and Fujifilm ED-530XT, Tokyo, Japan) by experienced endoscopists under fluoroscopic guidance. The therapeutic ERCP procedures were performed as standard of care following guideline recommendations, as follows: Biliary plastic stents in situ were retrieved using a snare or grasper. Proximally migrated biliary stents were pulled down using an extraction balloon or Dormia basket placed over the guidewire, which was advanced alongside the stent. Another technique for stent retrieval was grasping its distal end using a snare or grasper and directly pulling it out. Distally migrated biliary stents were retrieved using a snare or grasper when they were accessible. Inaccessible stents were followed up with abdominal X-ray. For patients with detected CBD stones, their removal was attempted using an extraction balloon or Dormia basket after extending the prior sphincterotomy and/or sphincteroplasty when required. Mechanical lithotripsy was used in the case of difficult stone extraction. If stone removal was incomplete, new plastic stents were inserted [8]. The endoscopic management of the forgotten-biliary-plastic-stent-related complications is demonstrated in Figure 1.

### 2.6. Definitions

A forgotten biliary stent is defined as having CBD stent placement for more than one year without retrieval. Proximally migrated biliary stents are considered if their distal end is not endoscopically detected at the duodenal papilla and the stent is visible in the biliary tree during fluoroscopic examination. Distal migration is confirmed when the stent is located below its original position or it is not detected on the endoscopic and fluoroscopic examination [14,15].

### 2.7. Clinical Outcomes

The primary outcomes were the detection of the biliary-plastic-stent-related complications (e.g., acute cholangitis, stent clogging, new stone formation, and proximal and distal biliary stent migration) and the endoscopic management success rate. The secondary outcomes included complications related to mortality, hospital stay, and the number of therapeutic endoscopic sessions (one or more than one session). In the patients who had CBD re-stenting, ERCP was scheduled for further management. The patients with gallbladder stones were referred to surgery.

### 2.8. Statistical Analysis

The statistical analysis was performed using Statistical Package for the Social Sciences (SPSS, version 24.0. IBM Corp, Armonk, NY, USA). Quantitative data were expressed as mean ± standard deviation (SD). Qualitative data were expressed as frequency and percentage. The following tests were conducted: Independent-samples *t*-test of significance was used when comparing between two means. Chi-square (X2) test of significance was used in order to compare proportions between two qualitative parameters. *p* value < 0.05 was considered significant.

## 3. Results

### 3.1. Patients’ Characteristics

During the study period, 1968 ERCP procedures were performed. Of them, 527 patients had biliary plastic stents removed within 3 months (group 1), and 50 patients had biliary plastic stents retrieved after one year of their deployment (group 2); both groups were included in the present study. Characteristics of the study patients are shown in Table 1. There were statistically significant differences between the two groups regarding the duration of biliary plastic stenting and presenting symptoms. Meanwhile, there were no significant differences regarding other parameters (e.g., gender, age, and indications of biliary stenting). In the current study, the main indication of the endoscopic biliary plastic stenting was irretrievable CBD stones in 422 patients, 80.08% (group 1), and in 39 patients, 78% (group 2). In group 2, the stents were planned to be removed within 3 months of insertion; however, 30 patients intentionally did not attend their scheduled appointment because of noncompliance with the endoscopist’s instructions. Other patients admitted to unawareness of the stent insertion (6 patients) or the need for its removal (8 patients). There was no apparent reason for the forgotten stents in six patients.

### 3.2. Biliary-Plastic-Stent-Related Complications and the Management of the Study Patients

The incidence of biliary-plastic-stent-related complications (e.g., acute cholangitis, stent clogging, distal stent migration, new CBD stone formation, and proximal stent migration) was significantly higher in group 2 than in group 1, as illustrated in Table 2. The endoscopic management modalities of the study patients were summarized in Table 2. The patients in group 2 required a significantly higher number of endoscopic sessions and hospital admissions than those in group 1. All the study patients had uneventful outcomes without any complication-related mortality.

### 3.3. Flow Chart of the Forgotten-Biliary-Plastic-Stent-Related Complications and Management

**Figure 2:**
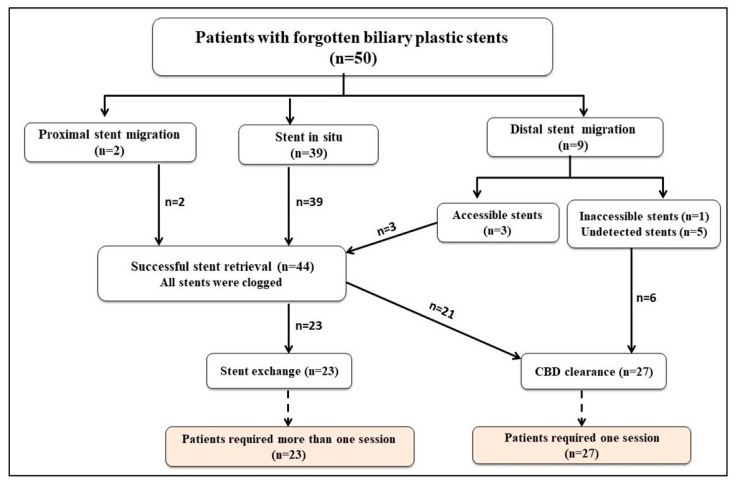
Flow chart of the forgotten-biliary-plastic-stent-related complications and management.

The flow chart revealed stent in situ (39 patients, 78%), distal stent migration (9 patients, 18%), and proximal stent migration (2 patients, 4%). Forty-four stents were retrieved (39 stents in situ, 3 accessible distally migrated stents, and 2 proximally migrated stents), and all of them were found to be clogged. Among these 44 patients, 23 patients had stent exchange, and 21 patients had CBD clearance. Furthermore, CBD clearance was achieved in the remaining 6 patients with distal stent migration (5 with undetected stents and 1 with an inaccessible stent) as illustrated in Figure 2.

### 3.4. Previous Studies and Case Reports of Forgotten Biliary Plastic Stents

The findings of previous studies and case reports of forgotten biliary plastic stents are summarized in Table 3.

## 4. Discussion

Biliary plastic stenting is a safe and broadly used treatment modality for short-term biliary decompression. However, long-term retained forgotten biliary stents can be hazardous. The forgotten biliary stents are often underestimated in clinical practice. Our study was performed to determine the forgotten-biliary-plastic-stent-related complications, their management, and the patients’ clinical outcomes.

Biliary plastic stents were deployed during the index ERCP for various benign biliary conditions. These stents were planned to be removed within 3 months of insertion to avoid possible complications related to prolonged stenting. These were in line with the studies of Perri et al. and Lawrence et al. [20,21].

In the current study, the main indication of biliary plastic stenting was irretrievable CBD stones in 422 patients, 80.08% (group 1), and in 39 patients, 78% (group 2). Our finding was in accordance with previous studies by Duman et al. and Taj et al.; they demonstrated that irretrievable CBD stones were the most common indication for biliary plastic stenting, accounting for 61% and 52.9% of their cases, respectively [14,22].

The mean duration of stent stay in patients with forgotten biliary plastic stents (group 2) was 29.09 ± 9.73 with a range of 13–46 months. It is worth noting that the most frequent reason for stents’ non-retrieval for extended periods is patients’ noncompliance with the endoscopists’ instructions. Patients’ unawareness of stent insertion or the need for its removal was another factor.

The incidence of biliary-plastic-stent-related complications (e.g., acute cholangitis, stent clogging, distal stent migration, new CBD stone formation, and proximal stent migration) was significantly higher in group 2 than in group 1. Acute cholangitis was found in 17 (34%) patients with forgotten biliary plastic stents. This is consistent with the findings of Duman et al. and Kumar et al.; they showed that cholangitis was frequently observed among their patients with higher percentages than ours (64.7%, and 66.6%, respectively) [15,16].

In patients with forgotten biliary stents, stent clogging was the most frequent encountered complication (44 patients, 88%), and this was in agreement with the work of Donelli et al., who showed that the major drawback of prolonged plastic stent placement was its tendency to clog [23]. Other complications included distal biliary stent migration (9 patients, 18%), new CBD stone formation (5 patients, 10%), and proximal biliary stent migration (2 patients, 4%). Duman et al. reported higher percentages of CBD stone formation (79%) and proximal stent migration (26.4%) in their patients than ours [15]. It was noted that 5 of 11 of our patients who previously had post-cholecystectomy biliary leak, Mirizzi syndrome, and CBD stricture were found to develop de novo CBD stones. In the remaining 39 patients who previously had irretrievable CBD stones, it was challenging to determine whether additional stones were newly developed alongside the previous ones or not. This could explain the lower percentage of new CBD stone formation in our study in comparison to those in other studies [15,16]. Therefore, new stone formation might be underestimated in the present study.

Stent clogging, CBD stone formation, and even acute cholangitis could be explained by duodenobiliary reflux, due to endoscopic sphincterotomy, and the retained biliary stent itself acting as a foreign body. These trigger bacterial colonization, biofilm formation, and calcium bilirubinate crystal deposition [24,25,26].

Nevertheless, two patients (4%) with forgotten biliary plastic stents were asymptomatic. This was consistent with the findings of Sohn et al., who stated that the biliary stent might remain in situ for years without causing any symptoms. Even a completely blocked stent might not disrupt bile flow because of the existence of additional pathways between the CBD wall and the stent [17].

Fortunately, in the present study, all biliary plastic stents and their related complications were successfully managed endoscopically, and there was no need for surgery or interventional radiology. This was in harmony with the finding of Duman et al. and Jaleel et al., who reported that all their patients were managed endoscopically [15,27]. On the other hand, surgical exploration for the management of stent-related complications was performed in 76.2% of the patients enrolled in Kumar et al.’s study [16]. The need for surgery could be attributable to the different complications encountered in their patients such as giant stentoliths and hepatolithiasis [16,18,28].

The laparoscopic cholecystectomy and CBD exploration are alternative treatments for choledocholithiasis in selected centers, particularly those with advanced laparoscopic surgeons. In this surgical intervention, a cholecystectomy and the removal of CBD stones are performed in a single step. Most importantly, sphincterotomy is avoided and there are no stents and possible further complications (e.g., acute pancreatitis, bleeding, and perforation) [29].

The limitations of the present study were its retrospective design. New CBD stone formation as a complication of forgotten biliary stents might have been underestimated in our study.

## 5. Conclusions

Based on this retrospective study, non-adherence to the endoscopists’ instructions is the main reason for retained biliary stents for a duration of more than one year. The forgotten stents had significantly higher complication rates, a higher number of endoscopic sessions, and a higher number of hospital admissions than those retrieved in accordance with the scheduled time. All patients were managed endoscopically with a technical success rate of 100%, with uneventful outcomes.

Recommendations: The patients who underwent ERCP with CBD stenting should be educated on having biliary stents, the scheduled date for stent removal, and the potential hazards of delaying removal. Giving appointment cards to the patients, as well as a short message service reminder, for scheduled stent removal is recommended to avoid stent-related undesirable outcomes. Calling patients that have not attended their scheduled appointments for stent removal is also considered. During ERCP reporting, we suggest to write the recommendation about biliary stent removal in both English and the patient’s native language to increase patients’ adherence to their subsequent ERCP appointments.

## Figures and Tables

**Table 1:** Characteristics of the study patients with biliary plastic stents (*n* = 577).

Parameters	Biliary Plastic Stent Removal (within 3 Months) *n* = 527		Biliary Plastic Stent Removal (after One Year) *n* = 50		*p* Value
	*N*	%	*N*	%	
Gender: male, female	278, 249	52.75%, 47.25%	32, 18	64%, 36%	0.127
Age (years), (mean ± SD)	51.76 ± 10.02		54.07 ± 7.70		0.113
Indications of biliary plastic stenting					
Irretrievable CBD stones	422	80.08%	39	78%	0.726
Post-cholecystectomy biliary leak	42	7.97%	5	10%	0.616
Mirizzi syndrome	48	9.11%	4	8%	0.794
Benign CBD stricture	15	2.85%	2	4%	0.860
Stent stay (months), (mean ± SD)	2.89 ± 0.31		29.09 ± 9.73		0.001 *
Presenting symptoms					
Jaundice	9	1.71%	46	92%	0.001 *
Abdominal pain	7	1.33%	33	66%	0.001 *
Fever	4	0.76%	17	34%	0.001 *
Asymptomatic	518	98.29%	2	4%	0.001 *

SD, standard deviation; CBD, common bile duct. *p* value < 0.05 is considered significant, * highly significant.

**Table 2:** Biliary-plastic-stent-related complications and the endoscopic management of the study patients (*n* = 577).

Parameters	Biliary Plastic Stent Removal (within 3 Months) *n* = 527		Biliary Plastic Stent Removal (after One Year) *n* = 50		*p* Value
	*N*	%	*N*	%	
Stent-related complications					
Acute cholangitis	4	0.76%	17	34%	0.001 *
Stent clogging	21	3.98%	44	88%	0.001 *
Distal stent migration	8	1.52%	9	18%	0.001 *
New CBD stone formation	0	0%	5	10%	0.001 *
Proximal stent migration	1	0.19%	2	4%	0.001 *
Endoscopic management					
Stent retrieval and CBD clearance	415	78.75%	21	42%	0.001 *
Stent retrieval and new stent insertion	108	20.49%	23	46%	0.001 *
CBD clearance in undetected/inaccessible stents	4	0.76%	6	12%	0.001 *
Endoscopic sessions					
One session	419	79.51%	27	54%	0.001 *
More than one session	108	20.49%	23	46%	0.001 *
Hospital stay	4	0.76%	17	34%	0.001 *

CBD, common bile duct. *p* value < 0.05 is considered significant, * highly significant.

**Table 3:** Findings of previous studies and case reports of forgotten biliary plastic stents.

Authors	Year	Study Design	Patients	Stent Stay	Main Indication	Most Common	Management
(*n*) of Stenting Complications							
Duman, A.E. [15]	2021	Retrospective	48	22.5 months	CBD stones	Biliary stone formation	Endoscopic
Kumar, S. [10]	2020	Case report	1	4 years	CBD stones	Giant Stentolith	Surgical
Kumar, S. [16]	2017	Retrospective	21	3.53 years	CBD stones	Acute cholangitis	Surgical/Endoscopic
Sohn, S.H. [17]	2017	Retrospective	38	22.6 ± 12.2 months	CBD stones	Acute cholangitis	Endoscopic
Barai, V. [18]	2016	Case report	1	102 months	Benign CBD stricture	Cholangitic liver abscesses	Surgical
Patel, T.J. [19]	2014	Case report	1	17 years	CBD stones	Perforated GB	Surgical

CBD, common bile duct; GB, gall bladder.

## Data Availability

Upon reasonable request, the data sets used in the current study are available from the corresponding author.

## Abbreviations

CBD	Common bile duct
ERCP	Endoscopic retrograde cholangiopancreatography
SPSS	Statistical Package for the Social Sciences
SD	Standard deviation
